# Juvenile Hormone Enhances Aversive Learning Performance in 2-Day Old Worker Honey Bees while Reducing Their Attraction to Queen Mandibular Pheromone

**DOI:** 10.1371/journal.pone.0112740

**Published:** 2014-11-12

**Authors:** H. James McQuillan, Shinichi Nakagawa, Alison R. Mercer

**Affiliations:** Department of Zoology, University of Otago, Dunedin, New Zealand; University of Arizona, United States of America

## Abstract

Previous studies have shown that exposing young worker bees (*Apis mellifera*) to queen mandibular pheromone (QMP) reduces their aversive learning performance, while enhancing their attraction to QMP. As QMP has been found to reduce the rate of juvenile hormone (JH) synthesis in worker bees, we examined whether aversive learning in 2-day old workers exposed to QMP from the time of adult emergence could be improved by injecting JH (10 µg in a 2 µl volume) into the haemolymph. We examined in addition, the effects of JH treatment on worker attraction to QMP, and on the levels of expression of amine receptor genes in the antennae, as well as in the mushroom bodies of the brain. We found that memory acquisition and 1-hour memory recall were enhanced by JH. In contrast, JH treatment reduced the bees’ attraction towards a synthetic strip impregnated with QMP (Bee Boost). Levels of expression of the dopamine receptor gene *Amdop1* were significantly lower in the mushroom bodies of JH-treated bees than in bees treated with vehicle alone (acetone diluted with bee ringer). Expression of the octopamine receptor gene, *Amoa1*, in this brain region was also affected by JH treatment, and in the antennae, *Amoa1* transcript levels were significantly lower in JH-treated bees compared to controls. The results of this study suggest that QMP’s effects on JH synthesis may contribute to reducing aversive learning performance and enhancing attraction to QMP in young worker bees.

## Introduction

In a honey bee colony, the queen bee advertises her presence using chemical signals known as pheromones [1], [2]. Queen mandibular pheromone (QMP), a diverse mixture of compounds secreted from the mandibular glands [2], ensures among other things that the queen is well attended by nurses and that young bees do not associate any negative experiences with the queen. Exposing young worker bees to synthetic queen mandibular pheromone (QMP) during their early adult life has been found to reduce aversive learning performance in young bees [3], [4]. QMP exposure has also been shown to alter amine receptor gene expression, including a reduction in the brain and antennal expression of the dopamine receptor gene *Amdop1* [5], [6], and reduced expression of the putative dopamine/ecdysone receptor gene *Amgpcr19* in the mushroom bodies (MBs) of the brain [4]. Early exposure to this pheromone also increases the likelihood that young bees will show attraction towards QMP [6]. The QMP-induced behavioural effects are known to be transient, but why they occur remains unclear. Here we investigate the possibility that this pheromone-induced behavioural plasticity may involve QMP-induced modulation of juvenile hormone (JH) titres in the bee. Levels of JH rise transiently between 1- and 4-days post adult emergence, depending upon the colony [7], [8]. During this transient rise JH titres reach a level that is approximately 1/10 of that detected in foragers [7], [9], [10]. JH titres influence the rate of worker bee behavioural development [7], [11], [12], [13], and one factor known to affect JH titres is QMP. QMP has been found to reduce the rate of JH synthesis in worker bees [14] and to lower the circulating titres of this hormone [15]. This raises the possibility that QMP’s effects on aversive learning performance, and the likelihood that young bees will be attracted to this pheromone, may be mediated via QMP’s effects on JH titres in the bee. To explore this possibility, we examined whether injection of JH into the haemolymph of 2-day old bees, maintained from the time of adult emergence in the presence of QMP, affected their aversive learning performance, or their attraction to QMP. As amine receptor genes have been implicated in the modulation of behavioural responses in insects, we in addition examined the effects of JH on the expression of amine receptor genes in 2-day old worker bees. Amine receptor gene expression levels were examined in the MBs, as these structures play a critical role in appetitive and aversive memory formation, reviewed by [16], [17], [18], [19]. We examined expression levels in the antennae also, since modulation at the level of these multifunctional organs has been implicated in shifts in worker bee attraction to QMP [6]. We demonstrate that JH not only alters the behaviour of young bees but also the MB and antennal expression of amine receptor genes, suggesting that QMP-induced changes in circulating JH titres are likely to contribute to the effects of this pheromone upon both learning behaviour and attraction of young bees to QMP.

## Materials and Methods

**Figure 1:**
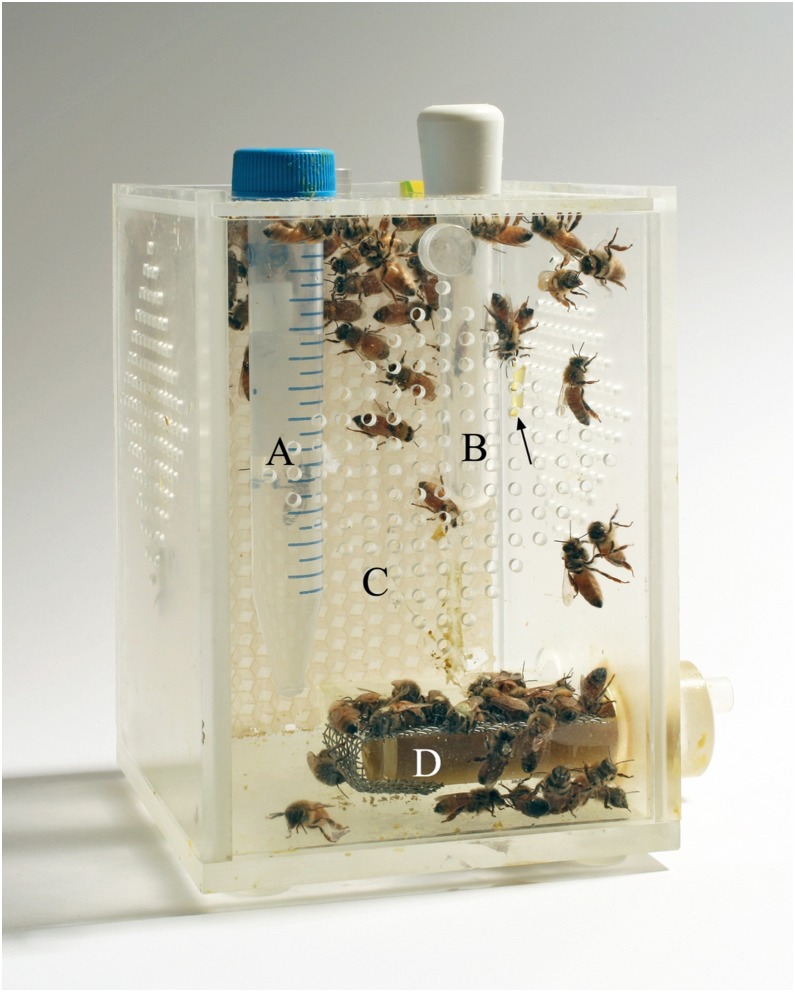
Cage containing bees exposed to QMP from the time of adult emergence. (A) Water feeder with small holes at the base. (B) Spare feeder. (C) Foundation comb that was replaced when cages were cleaned between sets of bees (D) Food feeder covered with a fine wire mesh to prevent bees from becoming coated with food. Arrow indicates a strip impregnated with QMP (Bee Boost) attached to the inside of the cage with a fine piece of wire. Cage dimensions (100/100/1350 mm).

All bees used in this study were sourced from colonies housed at the Department of Zoology, University of Otago. In order to obtain young bees of a known age, brood frames were sourced from several different colonies, with colony choice being dependent on available brood stocks. The brood frames were held in the laboratory in a humidified incubator at 34°C. Newly-emerged adults were typically collected within 1–2 hours of emergence and maintained along with a cluster of 50–60 sister bees (over night emergers) in acetate cages (Figure. 1), based on a design used by [20]. Bees were provisioned with a diet consisting of finely ground pollen mixed with honey in a 4∶1 ratio and a 30% sucrose solution added to form a moist paste. Using the methodology of [6], approximately two queen equivalents of synthetic QMP in the form of a synthetic blend in commercially available strips (Bee Boost, Phero Tech, Delta, BC, Canada) was included in all cages.

### JH treatment

Using 2-day old bees exposed to QMP from the time of adult emergence, we examined the effects of JH treatment upon aversive learning performance, worker attraction towards QMP, and amine receptor gene expression in the antennae and MBs of the brain. Prior to treatment with JH, or with vehicle only (controls), all bees were lightly cold anaesthetised, harnessed in chilled copper tubing and positioned in a microinjection apparatus. Injections were performed using a 5 µl Hamilton syringe (900 series, Sigma-Aldrich) equipped with a 26 s gauge needle which was inserted through the intersegmental membrane between the fifth and sixth tergal plates. JHIII was diluted in acetone as described by [21] to a concentration of 20 µg/µl and then 1∶4 in bee ringer (130 mM NaCl, 6 mM KCl, 4 mM MgCl, 5 mM CaCl, 160 mM sucrose, 25 mM glucose, 10 mM HEPES, pH = 6.7, osmolarity ≈500 mOsmol) to achieve a final concentration of 5 µg/µl. Each bee received an injection volume of 2 µl, achieving a final dose of 10 µg/bee. Controls for each experiment were injected with vehicle only (acetone diluted 1∶4 in bee ringer). To help prevent haemolymph loss, the needle was left in place for 15–30 seconds prior to removal. Any bees with obvious loss of haemolymph were not included. Following treatment with JH, or with vehicle alone, bees were left for 3 hours before testing their aversive learning performance, their attraction to QMP, or collecting MB and antennal samples to analyse gene transcript levels in these tissues. A treatment duration of 3 hours was chosen as earlier reports have shown that there can be significant effects on gene expression in honeybees 3 hours after treatment with JH [21]. Typically bees were treated in groups of 6–8 bees per day.

### Aversive learning

To examine aversive learning performance, JH-treated bees (n = 42), and bees treated with vehicle alone (n = 42) were transferred to harnesses for aversive conditioning. Differential aversive conditioning using the sting extension reflex was carried out as described by [22]. Briefly, bees received 12 trials delivered in a pseudo randomised order, six of which consisted of a 5 s puff of the floral odour, eugenol (conditioned stimulus CS+), which was reinforced in the final 2 s with a 7.5 V, 2 s electric shock. In the six remaining trials, bees were presented with a 5 s puff of 2-hexanol (CS−), which was not reinforced. To maintain a balanced presentation the first odour presented was alternated between the CS+ and CS−. An inter-trial interval of 10 minutes was used throughout. Learning acquisition curves were generated by recording whether or not bees displayed a sting extension response (SER) when presented with CS+ or CS−. In the case of CS+, responses were recorded prior to the application of the unconditioned stimulus (US, electric shock). One hour after the last conditioning trial, a memory retention test was performed in which the CS+ odour was presented without electric shock. Whether or not bees continued to display the sting extension reflex in response to the unconditioned stimulus (electric shock) was examined after the memory retention test. Any bees not showing the reflex response to the shock more than twice during conditioning or when tested following the retention test were routinely removed from the analysis. In the current study, no bees required removal from the analysis and only one control bee failed to respond to the shock on a single occasion.

**Table 1:** *F* values and degrees of freedom (shown in brackets) obtained by two-way ANOVA of the behaviour of 2-day old QMP-exposed control and JH-treated bees towards a control strip or QMP strip.

	Mean number of teststrip contacts/min	Average area occupied(cm^2^)
Treatment	_(1,92)_ 8.871	_(1,92)_ 0.429
Strip	_(1,92)_ 3.785	_(1,92)_ 9.108
Interaction	_(1,92)_ 3.143	_(1,92)_ 1.482

### Responsiveness to stimulation with electric shock

In order to determine if treatment with JH affects a bee’s responsiveness to electric shock, the responses of JH-treated bees to a series of electric shocks of increasing intensity were assessed and compared with the responses of bees treated with vehicle alone (controls). Controls and JH-treated bees were prepared as described earlier and harnessed in holders used for aversive learning. Electric shocks of increasing intensity (0.25, 0.5, 1, 2, 4 and 8 V) were delivered to each bee and the bees’ responses were recorded. To monitor for changes in behaviour resulting from placement in the experimental set up, placement trials were interspersed between each test trial. In placement trials, bees were placed in the set up, but no electric shock was delivered. An inter-trial interval of 2 minutes was maintained throughout.

### QMP attraction assay

**Figure 2:**
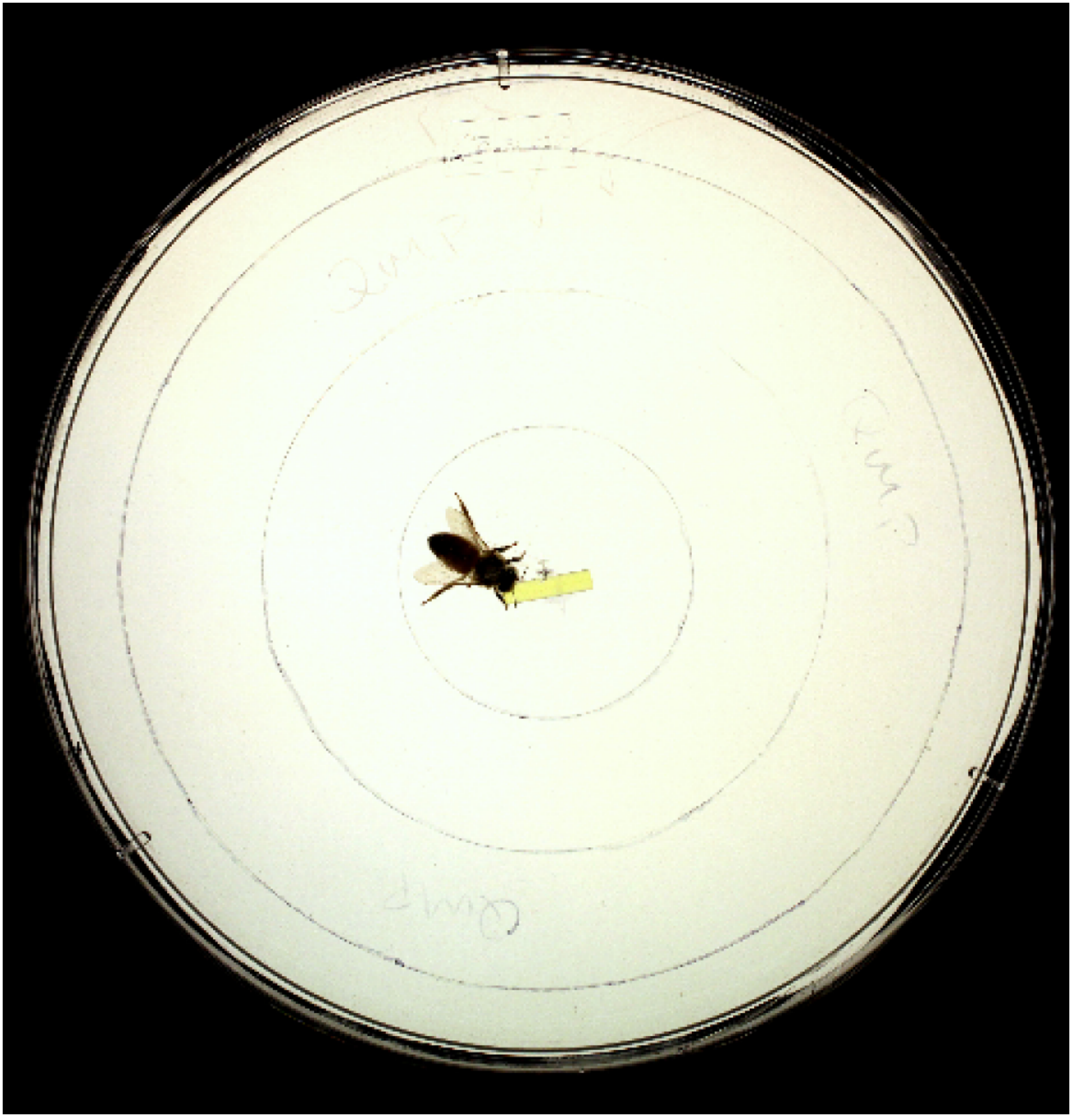
Photo of a QMP attraction assay test arena. A 2-day old bee can be seen contacting the QMP strip located at the centre of the arena.

A previously described protocol [6] was employed to assess attraction to QMP in controls (n = 25) and JH-treated bees (n = 23). Three hours following treatment, the attraction displayed by JH-treated bees towards a 1 cm QMP-impregnated strip (Bee Boost) containing approximately two queen equivalents versus a control strip of the same dimensions containing no QMP was assessed and compared to the attraction displayed by controls. The assay was performed using an inverted Petri dish placed on an overhead transparency sheet on top of a light box. Different arenas were used to test responses to the QMP strip and the control strip and between trials, test arenas and control strips were cleaned with 70% ethanol. The order of testing was randomised by alternating the first test between the QMP and control strip. Each bee was allowed to acclimatise to the test arena for one minute and then over a period of four minutes the number of body contacts the bee made with the QMP strip, or control strip, was recorded. In addition, concentric circles were used to subdivide each arena into four sectors (Figure 2) and the position of the bee within the arena was recorded every 20 seconds. The mean area occupied by each bee during the four minutes was calculated by multiplying the number of counts in each sector by the sector area divided by the total number (13) of observations. A bee that spent more time in the centre of the dish, close to the QMP strip or control strip, would register a lower mean area occupied than a bee that spent most of the time at, or near, the periphery of the arena, see [6]. The percentage of total counts in each sector was divided by the sector area and plotted against distance from the centre of the dish in order to provide graphical representation of the proximity of bees to the QMP strip or the control strip during the observational period. QMP attraction assays were performed between 2–4 hours following treatment.

### Measurement of gene transcript levels using real-time qPCR

Bees treated with JH (n = 5) or with vehicle alone (n = 5) were used to examine the effects of JH on amine receptor gene expression in the antennae and MBs of the brain. Five amine-receptor genes were analysed; the dopamine receptor genes, *Amdop1*, *Amdop2* and *Amdop3*, the octopamine receptor gene, *Amoa1,* and the honey bee orthologue (*Amgpcr19*) of the *Drosophila* dopamine/ecdysone receptor gene, DmDopEcR [23]. Bees were cold immobilised prior to the collection of MB and antennal tissue. MB calyces were promptly plucked from both hemispheres of the brain using fine forceps, frozen in 1.5 ml tubes on dry ice and stored at −80°C until further processing. Antennal samples were collected 3 hours post treatment, as described previously [24]. Total RNA was extracted from MB calyces and antennae by homogenisation of samples in Trizol reagent (Invitrogen, Carlsbad, CA) prior to isolation using PureLink Mini Total RNA purification columns (Invitrogen). Real time qPCR analysis performed as described elsewhere [24], [25]. Briefly, 50 ng of MB or antennal RNA was reverse transcribed using VILO Superscript (Invitrogen, Carlsbad, CA). Gene-specific amplification products were generated with ExpressSYBR GreenER qPCR SuperMix (Invitrogen, Carlsbad, CA) using an MX3000P instrument (STRATAGENE, La Jola CA). Gene transcript levels were normalised using the geometric mean of *Rpn2* and *Rps8* as this was found to be a stable combination of reference genes for MB samples (see Results section) as well as for antennae (Figure S1). Primer details and assay properties are described elsewhere [4], [25].

### Statistical analysis

Generalised linear mixed-effects models, GLMMs (binomial error structure with the logit-link function) using the *lmer* function were used for analysing aversive learning data, recorded either as 0 (no response), or 1 (SER). This method is recommended by [26] for the analysis of categorical data. GLMMs enabled comparisons of the slopes of response curves for CS+ and CS− in different treatment groups, with trial numbers and treatments as fixed factors. Bee identities and session identities (CS+ and CS−) were included as random factors along with trial numbers in a session as random slopes. We then used generalised linear models, GLM (binomial error structure with the logit-link function) using the R function *brglm* in the package *brglm* [27] to compare percentages of control bees versus JH-treated bees displaying sting extension in response to CS+ during the retention tests. The *brglm* function uses a bias-reduction method, which deals with no responses in particular groups. For LMMs, it is not straightforward to obtain the degrees of freedom (df) required for calculating *P* values [28]; note that binary GLMMs do not require df because of the use of z values. For this study, we used the scheme by [29], where df for LMMs is obtained by subtracting levels of random effects from the total observations.

Differences between amine receptor gene expression levels in control and JH treated groups were analysed using two-tailed unpaired t-tests performed in PRISM (GraphPad Software Inc, San Diego, CA). This software was also used to examine differences between the number of test strip contacts/min in control and JH treated 2-day old bees by two-way ANOVA, followed by Tukey’s multiple comparison tests. All other statistical analyses were conducted in the R environment [30].

## Results

### JH improves aversive learning performance in 2-day old bees

**Figure 3:**
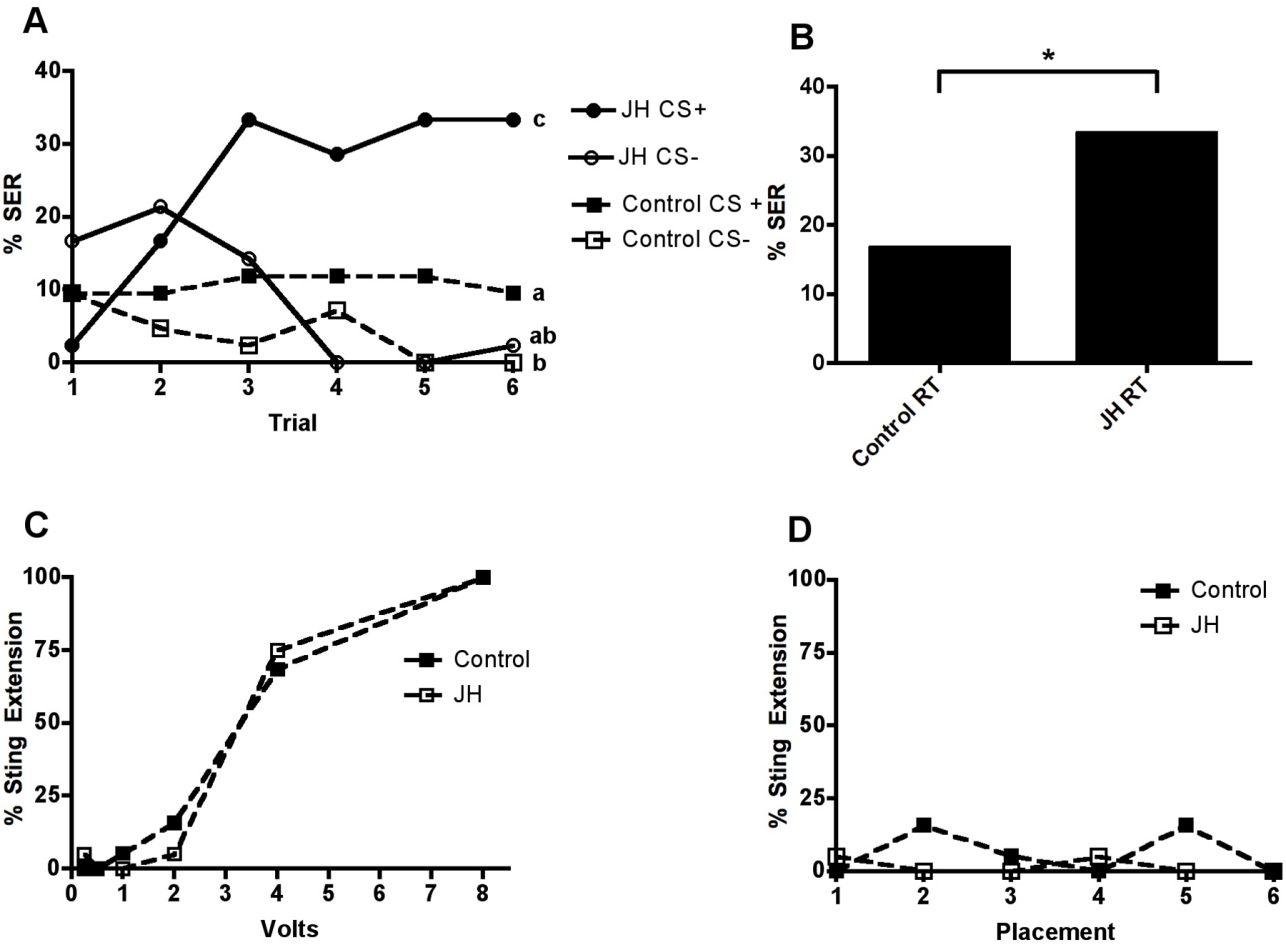
Effects of JH on aversive learning (A) and memory recall (B) in 2-day old worker bees raised with QMP. Learning performance in control bees injected with vehicle alone (n = 42) was compared with learning in bees treated with JH (n = 42). Bees were conditioned using differential conditioning of the sting extension reflex. Each bee received 12 pseudo-randomised conditioning trials. In six trials, eugenol was paired with an electric shock (CS+) in the remaining trials 2-hexanol was presented without reinforcement (CS−). (A) The percentage of control bees displaying a sting extension response (SER) to CS+ did not change significantly across successive trials (GLMM, *P = *0.7355, z = −0.338). However, the percentage of bees displaying sting extension in response to the CS− odour significantly decreased (GLMM, *P* = 0.0258, z = −2.228). The slopes of the CS+ and CS− response curves of the control bees were significantly different (GLMM, *P*≤0.05). In contrast to controls, JH-treated bees showed clear evidence of learning with a significant increase in the % of bees displaying SER over successive conditioning trials with CS+ (GLMM, *P*≤0.001, z = 3.784). These bees also displayed a significant decrease in responses to CS− (GLMM, *P*≤0.001, z = −3.964). The slopes of the CS+ and CS− response curves of the JH treated bees were also significantly different (GLMM, *P*≤0.001). Significant differences in responses to CS+ and CS− are indicated by plots that do not share a letter (GLM, *P*≤0.01). (B) The percentage of bees responding to CS+ with sting extension during the retention test was significantly higher in JH-treated bees than in controls, as indicated by an asterisk (*P*≤0.01). (C) Percentage of bees responding to electric shock at intensities ranging between 0.25 and 8 volts. Responses of control bees injected with vehicle alone did not differ from those of JH treated bees at any of the voltages tested. (D) Neither group of bees responded significantly with sting extension in response to placement alone.

Bees maintained until 2-days of age with QMP and injected with vehicle alone showed little evidence of aversive learning (Figure 3A). While there was a significant decrease in the percentage of bees responding to the non-reinforced odour (CS−, *P* = 0.0258), the slope of the acquisition curve for the reinforced (CS+) odour was not significantly different from zero (*P* = 0.7355). In contrast, bees of the same age treated with JH displayed clear evidence of aversive learning. Over successive conditioning trials, the percentage of JH-treated bees exhibiting sting extension when presented with the CS+ odour increased and the slope of the CS+ acquisition curve was significantly greater than zero (*P*≤0.001). As in controls, the percentage of bees displaying sting extension in response to CS− declined and the slope of the CS− curve was significantly less than zero (*P*≤0.001). In the retention test, one hour following the final conditioning trial, the percentage of bees displaying sting extension in response to CS+ in the JH-treated group was significantly higher than in the control group (*P* = 0.0045, Figure 3B). It should be noted that responsiveness to electric shocks did not differ between the control and JH groups or change during either conditioning or following the retention test. Furthermore, responsiveness to electric shocks of increasing intensity, or to placement in the testing station, did not differ between control- and JH-treated bees (Figure 3C, D).

### Following treatment with JH, two-day old bees were not attracted to QMP

**Figure 4:**
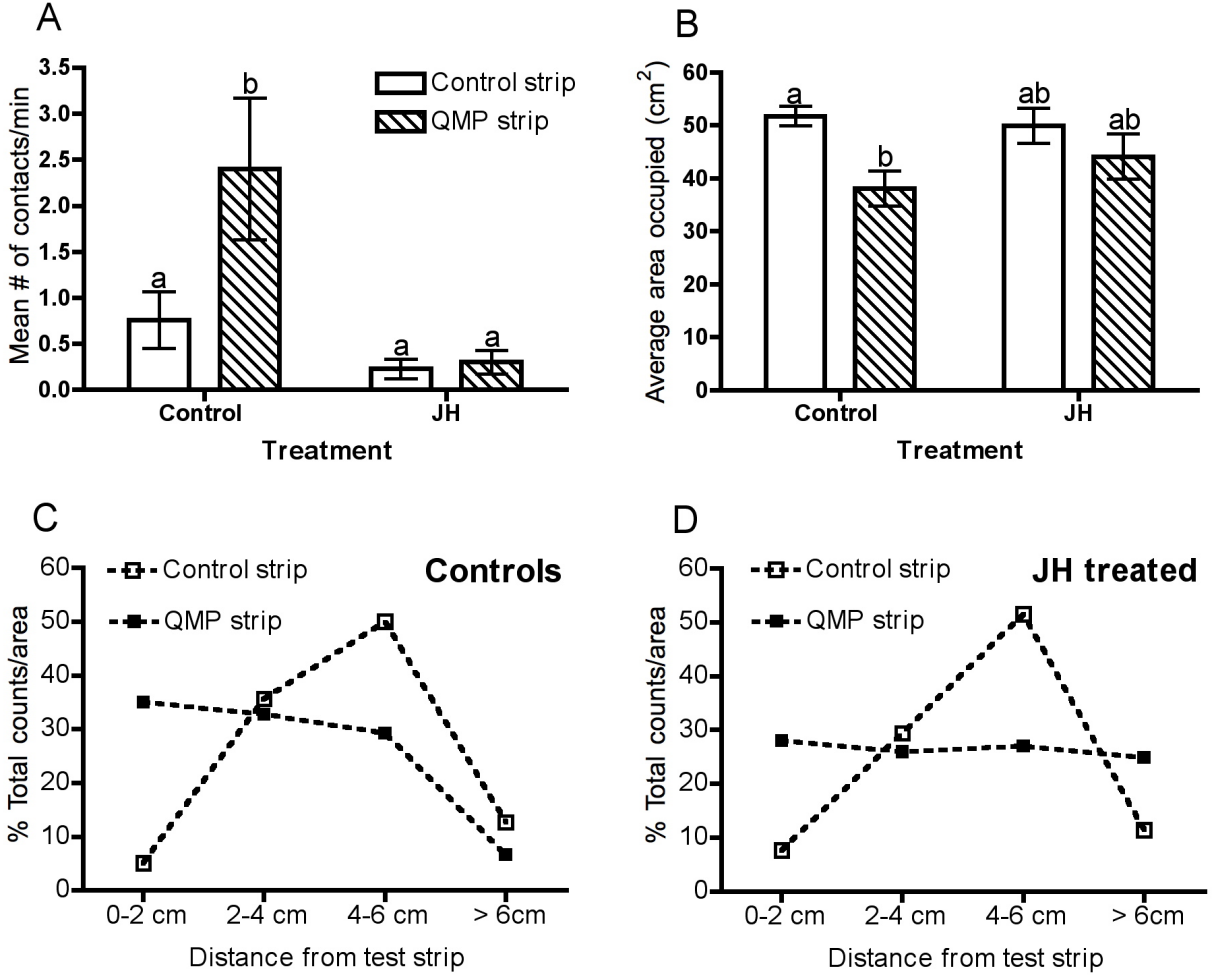
Behaviour of 2-day old controls (n = 25) and JH-treated bees (n = 23) towards a control strip versus a QMP strip. (A) Mean number of contacts/min with the test strip (2-WAY ANOVA, treatment, *P* = 0.0037, test strip, *P* = 0.0548, interaction, *P* = 0.0796). (B) Average area occupied (cm^2^) during the four minute test period when in the presence of either a control strip or a QMP strip (2-WAY ANOVA, treatment, *P* = 0.0033, strip, *P* = 0.5142, interaction, *P* = 0.2266). The percentage of total counts/area for control (C) and JH treated (D) bees in the presence of either a control strip or a QMP strip. F values and degrees of freedom are provided in Table 1.

Attraction to QMP was assessed in bees treated with the vehicle alone (controls) and in bees treated with JH (Figure 4A). The number of contacts made with a test strip was clearly influenced by the presence or absence of QMP in the strip (*P* = 0.0548) and the number of contacts was altered significantly by treatment with JH (*P* = 0.0037). Control bees showed significantly greater attraction towards the QMP-impregnated strip than the control strip (*P*<0.05), whereas bees treated with JH made relatively few contacts with either the control strip or the QMP-impregnated strip (Figure 4A).

Differing responses to the control strip and the QMP strip were apparent also in the area bees occupied in the arena (Figure 4B). While the average area of the arena occupied was influenced significantly by the presence of QMP (*P* = 0.033), it was not affected by treatment with JH (*P* = 0.5412). When in the presence of the QMP strip, control bees occupied a significantly smaller area of the arena (P<0.05), indicating that they remained in closer proximity to the QMP strip. In contrast, JH-treated bees occupied similar areas of the arena irrespective of whether the control strip or the QMP strip was located in the centre.

### JH treatment altered gene transcript levels in the MBs and antennae

**Figure 5:**
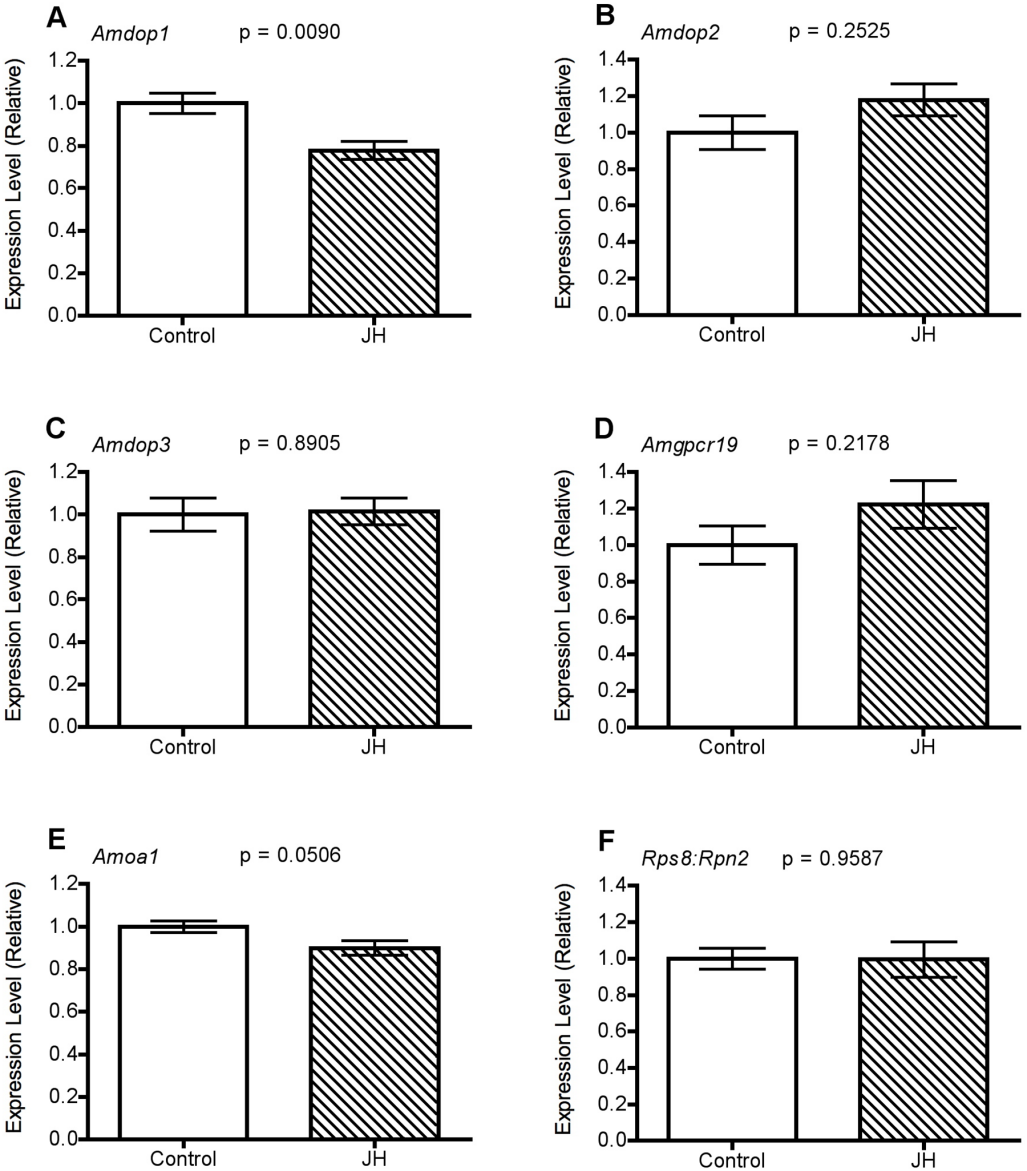
Amine receptor gene expression in the MB of 2-day old bees 3-hours following treatment with vehicle only (Control) or JH. All bees were exposed to QMP from the time of adult emergence. Statistics: (two-tailed t-test) (A) *Amdop1* (t_(8)_ = 3.033) (B) *Amdop2* (t_(8)_ = 1.091) (C) *Amdop3* (t_(8)_ = 0.3248) (D) *Amgpcr19* (t_(8)_ = 1.732) (E) *Amoa1* (t_(8)_ = 2.299). Expression levels were normalised using the geometric mean of *Rpn2* and *Rps8* (F) (t_(8)_ = 0.1073).

**Figure 6:**
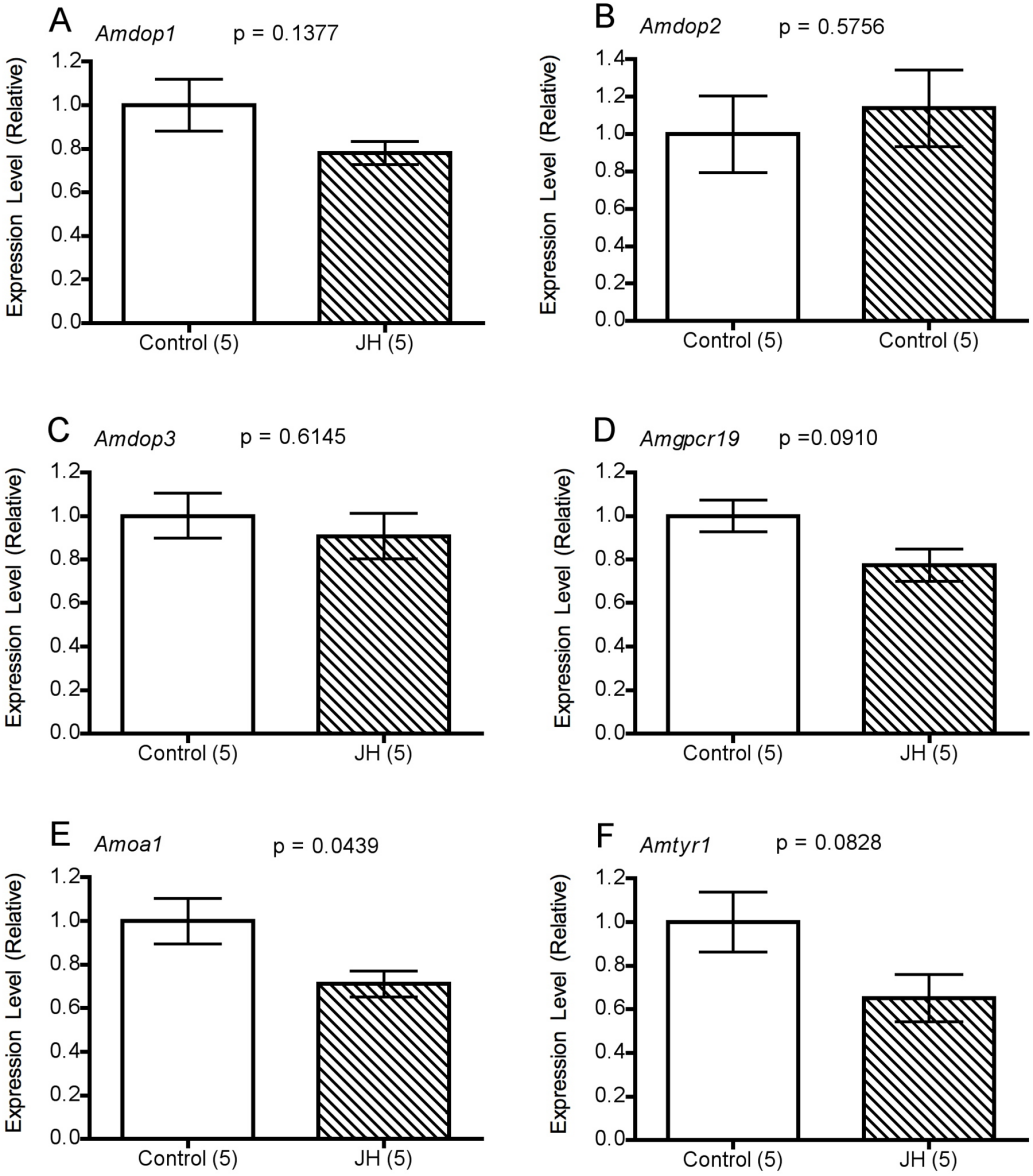
Amine receptor gene expression in the antennae of 2-day old bees 3-hours after treatment with vehicle alone (Control) or JH. Expression levels were normalised using the geometric mean of *Rpn2* and *Rps8*. Statistics (A) *Amdop1* (*t_8_* = 1.649; *P = *0.1377) (B) *Amdop2* (*t_8_ = *0.5836) (C) *Amdop3* (*t_8_* = 0.5239) (D) *Amgpcr19* (*t_8_* = 1.921) (E) *Amoa1* (*t_8_* = 2.389) (F) *Amtyr1* (*t_8_* = 1.982).

For the majority of genes examined, transcript levels were not altered significantly by treatment with JH (Figures 5,6). However, in the MBs levels of Amdop1 transcript were found to be significantly lower in JH-treated bees than in controls (P = 0.0090, Figure 5A). A similar trend was apparent for Amoa1 (P = 0.0506, Figure 5E), and in antennal samples, Amoa1 transcript levels were significantly lower in JH-treated bees than in controls (P = 0.0439, Figure 6E). While Amgpc19 and Amtyr1 transcript levels in the antennae were slightly lower also in JH-treated bees than in controls, the effects of JH treatment on the level of expression of these genes were not statistically significant (Figure 6D, F).

## Discussion

QMP is a multicomponent pheromone that has wide-ranging effects on the behaviour and physiology of worker bees [1], [2]. Among its many effects, QMP has been found to reduce aversive learning performance in young (4- and 6-day old) worker bees [3], and to increase their attraction to this critically important pheromone [6]. QMP has long been known to reduce the rate of JH synthesis [14], and to lower the levels of this hormone in the haemolymph of young bees [15]. The results that we have presented in this paper indicate that QMP-induced changes in circulating JH titres are likely to contribute to the effects of this pheromone not only on the learning behaviour of young worker bees, but also their attraction to QMP. We have shown that aversive learning performance in 2-day old workers, exposed to QMP from the time of adult emergence, can be improved by injecting JH into the haemolymph of the bees. These improvements in learning performance are not attributable to JH-induced changes in responsiveness to electric shock because we found that in controls and JH-treated bees, levels of responsiveness to electric shock stimuli were very similar.

Increasing JH levels in the haemolymph also reduced the likelihood of two-day old bees showing attraction to QMP, despite their early exposure to this pheromone.

As shown previously [6], 2-day old bees exposed to QMP displayed greater attraction towards a synthetic strip of QMP than controls. However, when bees were treated with JH we observed a marked reduction in the level of attraction towards the QMP-impregnated strip. This was evident in the reduced number of contacts with the QMP strip, and also in the greater proportion of time JH-treated bees spent at distance of >6 cm away from this strip. The responses of JH-treated 2-day old bees resemble those described previously for foragers [6].

Strong attraction to QMP has been associated with high levels of *Amoa1* expression in the antennae of 2-day old bees [6], suggesting that worker attraction to QMP may be peripherally modulated. Consistent with this hypothesis, we show in this study that JH-induced reduction of worker bee attraction to QMP is associated also with a significant down-regulation in *Amoa1* transcript levels in the antennae of JH-treated bees. We have described age-related changes in the levels of expression of this same gene, and changes also in QMP attraction with age. For example, *Amoa1* expression is lower in the antennae of 6-day old workers than in pollen forager bees, and lower also in precocious foragers than in bees of the same age performing tasks within the colony [24]. When bees of foraging age are placed in an arena with a strip impregnated with QMP, unlike 2-day olds they generally avoid contact with the QMP strip [6]. In male moths, the biogenic amine octopamine has been shown to affect responses to female sex pheromone [31], [32], [33], and there is strong evidence that these effects occur, at least in part, through the peripheral actions of this amine [34], [35], [36], [37], [38]. However, central effects of JH and octopamine have also been demonstrated in moths, as well as in bees. For example, in male *Argotis ipsilon*, JH accelerates the maturation of antennal lobe neurons, and the sensitivity of these neurons to female sex pheromone is increased by octopamine [39]. Recent evidence reveals that octopamine also modulates the activity of neural networks in the antennal lobes of honey bees [40].

In 2-day old bees also, effects of JH treatment were clearly not restricted to the periphery. Our results revealed lower levels of *Amoa1* transcript in the MBs of JH-treated bees than in controls. This is intriguing, as OA has been shown to impair avoidance learning in the bee [41]. A reduction in AmOA1 receptors could potentially contribute therefore, to improvements in aversive learning performance such as those observed in this study. Reduced expression of *Amdop1* in the MBs of JH-treated bees, however, is harder to reconcile with the observed enhancement of aversive learning and memory recall observed in 2-day old JH-treated bees, as the AmDOP1 receptor is believed to play a central role in aversive learning. Abnormal expression of the *Drosophila* ortholog of *Amdop1* (dDA1) in MBs of the fly, for example, has been shown to impair both appetitive and aversive learning [42], [43]. We have previously described age-related changes in the expression of amine receptor genes in the brain, including a reduction in the expression of *Amdop1* between emergence and 15-days of age, with some evidence of an age related decrease also in the expression of *Amoa1* [25]. However, the functional significance of such changes has yet to be determined.

Whether the changes in gene expression following treatment with JH observed in this study contribute to improving aversive learning behaviour in 2-day old bees, or reducing their attraction to QMP is unclear. However, the results of this study strongly suggest that effects of this pheromone on circulating JH levels are likely to contribute to QMP-induced reductions in aversive learning performance in young worker bees, and enhanced attraction to QMP.

## Supporting Information

Figure S1 **Expression levels of the geometric mean of** ***Rpn2*** **and** ***Rps8*** **in the antennae of 2-day old bees 3-hours after treatment with vehicle alone (Control) or JH.** This stable combination of genes (*t_8_* = 1.649) was used as a reference to normalise amine receptor gene expression levels.
(TIFF)
