# Supplementary figures and images for: Juvenile Hormone Enhances Aversive Learning Performance in 2-Day Old Worker Honey Bees while Reducing Their Attraction to Queen Mandibular Pheromone
Source: PLoS One. 2014 Nov 12;9(11):e112740. doi: 10.1371/journal.pone.0112740 (PMC4229236; doi:10.1371/journal.pone.0112740)

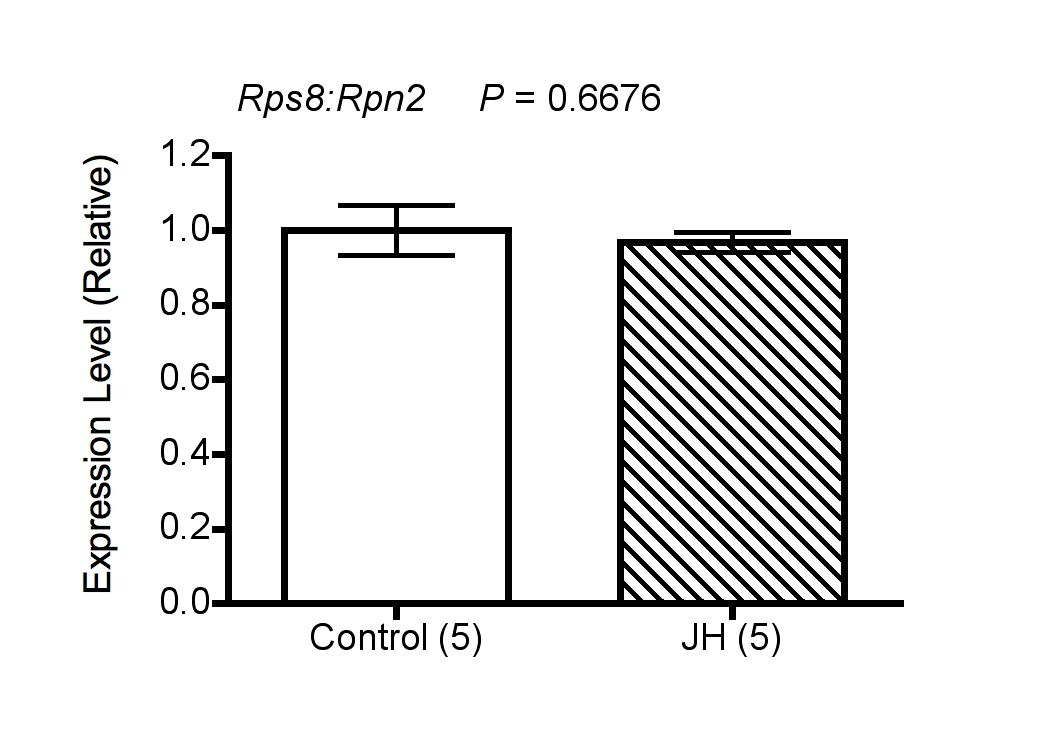

Supplement: Figure S1 — Expression levels of the geometric mean of Rpn2 and Rps8 in the antennae of 2-day old bees 3-hours after treatment with vehicle alone (Control) or JH. This stable combination of genes (t8 = 1.649) was used as a reference to normalise amine receptor gene expression levels. (TIFF) [file pone.0112740.s001.tiff]
